# Epilepsie révélant une chorée-acanthocytose: à propos d’un cas

**DOI:** 10.11604/pamj.2016.24.172.9686

**Published:** 2016-06-29

**Authors:** Nawfal Doghmi, Abdelghafour Elkoundi, Amine Meskine, Aziz Benakrout, Abdelouahed Baite, Cherqui Haimeur

**Affiliations:** 1 Service de Réanimation Médicale, Pôle Anesthésie-Réanimation, Hôpital Militaire Med V, Rabat, Maroc

**Keywords:** Chorée-acanthocythose, épilepsie, psychiatrique, Choreo-acanthocytosis, epilepsy, psychiatric

## Abstract

La chorée-acanthocytose (ChAc) est une affection autosomique récessive très rare causée par des mutations dans le gène VSP13A sur le chromosome 9q21. Elle est caractérisée par des signes neurologiques, des manifestations psychiatriques et une atteinte multisystémique comportant une myopathie, une neuropathie axonale et la présence de globules rouges spiculées ou acanthocytes. Rarement, l’épilepsie peut être le symptôme initial chez ces patients, ce qui peut considérablement retarder le diagnostic. Nous décrivons le cas d’un patient atteint de cette pathologie qui a présenté des crises épileptiques plusieurs années avant l’apparition des manifestations typiques.

## Abstract (en)

Chorea-acanthocytosis (ChAc) is an extremely rare autosomal recessive disorder caused by mutations in the VSP13A gene on chromosome 9q21. It is characterized by neurological symptoms, psychiatric manifestations and multisystem involvement resulting in myopathy, axonal neuropathy and presence of spiculated red blood cells or acanthocytes. Rarely, epilepsy may be the early symptom in these patients. This can lead to serious delays in diagnosis. We here report the case of a patient with this disease who had seizures several years before the onset of typical manifestations.

## Introduction

La chorée-acanthocytose (ChAc) est une maladie neurodégénérative très rare avec une expression clinique polymorphe, associant des manifestations neurologiques, hématologiques et systémiques et d’évolution progressive. L’épilepsie est rarement rapportée au cours de cette affection [1]. Nous rapportons ici une observation où l’épilepsie fut révélatrice d’une ChAc.

## Patient et observation

Il s’agit d’un patient âgé de 38 ans, issu d’un mariage consanguin du 1er degré et l’ainé d’une fratrie de 2 frères et une sœur bien portants. Le patient aurait présenté des crises épileptiques dans son enfance. Il fut également suivi pour un diabète insulino-requérant. L’anamnèse révéla l’apparition récente de mouvements anormaux buco-faciaux, un amaigrissement associé à une dysphagie modérée ainsi que des troubles périodiques de comportement. Le jour de son admission, Il présenta une crise convulsive tonico-clonique généralisée suivie d’une perte de conscience. A son admission, le patient était confus présentant des mouvements anormaux involontaires, brusques, désordonnés de type choréiques prédominant à la face avec protrusion de la langue. L’examen de la sphère buccale a objectivé des lésions récentes de la langue et de la lèvre inférieure compatibles avec des morsures. L’examen neurologique a trouvé des réflexes ostéotendineux abolis aux quatre membres sans déficit moteur ni troubles sensitifs. L’EEG a montré un ralentissement modéré de l’activité de fond avec des pointes d’ondes généralisées paroxystiques. La TDM ainsi que l’IRM cérébrale étaient sans anomalies. La recherche d’acanthocytes dans le sang périphérique était positive à 10%. La fonction rénale, hépatique et thyroïdienne étaient dans les limites de la normale. L’électrophorèse des lipides du patient était sans anomalie et l’étude de groupe Kells était négative. Le taux de CPK était élevé à 1500 UI/l et le taux de LDH était à 650 UI/l. L’électrocardiogramme ainsi que l’échocardiographie n’ont pas révélé de troubles et l’examen du fond d’œil était sans particularités. L’EMG a montré une réduction de l’amplitude des potentiels d’action sensitifs compatible avec une neuropathie sensitive de type axonal. Le diagnostic de ChAc a été établi sur des arguments anamnestiques et clinico-biologiques et un traitement associant valproate de sodium (500 mg x 2/j) et halopéridol (5 mg/j) a été initié. L’évolution s’est faite vers la régression des mouvements choréiques et le patient n’a pas présenté de crises épileptiques depuis.

## Discussion

La neuroacanthocytose est un groupe de maladies neurologiques associées à la présence dans le sang circulant de globules rouges de forme anormale spiculée ou acanthocytes [2]. Ce groupe nosologique comprend la ChAc, l´abêtalipoprotéinémie et le syndrome de MacLeod. La ChAc est une maladie génétique très rare, d´hérédité autosomique récessive, dont le gène VPS13A est identifié sur le chromosome 9 en région 9q21 [3] encodant pour la choréine. On estime le nombre de sujets atteints de cette pathologie à environ mille dans le monde [4]. Notre patient est le seul individu atteint dans sa famille. Ce qui est compatible avec une forme sporadique de la maladie déjà rapportée par Kuroiwa et al [5]. De plus, vu qu’il est issu d’un mariage consanguin du 1er degré, on pourrait avancer l´hypothèse d´une transmission autosomique récessive, et seule l´apparition de nouveau cas, la recherche d´acanthocytes chez les autres membres de la famille avec une étude génomique permettront de remettre en question cette hypothèse. Cliniquement, cette affection se caractérise d´abord par une association de mouvements anormaux intéressant principalement la région bucco-faciale et empruntant des symptômes à la chorée de Huntington et au syndrome de Gilles de la Tourette: dyskinésies, dystonies, mouvements choréiformes et tics [2, 3, 6]. Les patients ont des mouvements bucco-faciaux incessants, associés à une dysarthrie, et à des bruits surajoutés (claquement de langue, bruits de succion, vocalisations involontaires exceptionnellement). Ces mouvements peuvent avoir des conséquences graves, notamment des morsures mutilantes de la langue ou des lèvres (le très classique « lip-biting » anglo-saxon qui est plus spécifique de la ChAc), ou une dysphagie fonctionnelle secondaire aux dyskinésies linguales. On observe également des mouvements choréiques des membres, prédominant aux membres inférieurs. Il peut apparaître secondairement un syndrome parkinsonien, qui succède à la disparition des mouvements anormaux (probablement par épuisement des récepteurs dopaminergiques après hyperstimulation) [2, 6]. Dans la plupart des cas, les crises épileptiques surviennent au cours de la maladie à côté des autres manifestations. L’épilepsie comme symptôme initial précédant l’apparition des mouvements involontaires a été très rarement rapportée dans la ChAc [7–8]. Le délai moyen avant la découverte était de 10 ans dans la série de Alasmi et al. [9] ce qui pourrai faire errer le diagnostic pendant des années comme était le cas chez notre patient. Dans un tiers des cas, ce sont des crises généralisées à point de départ temporal [10, 11]. La physiopathologie des crises épileptiques au cours de la ChAc n’est pas encore élucidée. Une hypoperfusion et une réduction de la consommation d’oxygène et du métabolisme du glucose au niveau du noyau caudé, du noyau postérieur du putamen et au niveau du cortex frontal ont été constatées chez ces patients [12, 13]. La TDM et l’IRM cérébrale montre souvent une atrophie du striatum et du noyau caudé avec un hypersignal T2 [8, 14]. Un substratum anatomique pouvant expliquer la survenue des crises épileptiques au cours de la ChAc n’est pas connu à ce jour d’autant plus qu’aucune atteinte corticale cérébrale n’a été rapportée.

À ce tableau, s´ajoutent des troubles neuropsychologiques et comportementaux: troubles anxieux, obsessionnels compulsifs, dépressifs. Ces troubles s´expliquent par un dysfonctionnement sous-cortico-frontal qui parfois évolue vers une démence. Une atteinte neuromusculaire complète le tableau dans 62% des cas et peut affecter aussi bien le nerf que le muscle. Il s´agit d´une myopathie dans 60 % des cas [2]. Elle est symétrique dans la ChAc. La biopsie musculaire peut être normale ou montrer une atrophie des fibres musculaires avec centralisations nucléaires. Lorsqu´il s´agit d´une neuropathie, elle est à prédominance sensitive, de type axonale et peut dans la moitié des cas ne se manifester que par une aréflexie tendineuse. Une atteinte cardiaque à type de cardiomyopathie ou d’une hypertrophie ventriculaire gauche est rarement rapportée. La présence d´acanthocytes dans le sang circulant permet de porter le diagnostic. Cette recherche doit être demandée au biologiste par l´analyse du frottis: un examen de routine automatisé ne permet pas de les mettre en évidence. L´examen devra être répété et au besoin sensibilisé par le biologiste en cas de négativité. Le taux est considéré positif à partir de 3 à 5 % [2] et il n´existe pas de corrélation entre le taux d´acanthocytes et la sévérité de la maladie. La seconde étape est de faire un phénotypage du groupe sanguin Kell, surtout lorsqu´il y a possibilité d´une transmission liée au chromosome X, afin d´éliminer un phénotype de type Macleod. Le taux des créatine-phosphokinases est élevé dans la moitié des cas. La biopsie musculaire est aspécifique mais la tomodensitométrie musculaire peut orienter le diagnostic en précisant la sélectivité du déficit musculaire: une atteinte asymétrique évoque plutôt un syndrome de Macleod. D’autres diagnostics différentiels sont à éliminer. L’abétalipoprotéinémie a été écarté chez notre patient par la normalité de l’électrophorèse des lipides et l’absence d’une rétinite pigmentaire au fond d’œil. L’absence de tics moteurs élimine la maladie de Gilles de la Tourette. La présence d’acanthocytes peut être observée également en cas de malabsorption des lipides, de cirrhose alcoolique, des dysthyroïdies, d’anorexie mentale et chez les splénectomisés, ce qui n’est pas le cas de notre patient. L’expérience avec les thérapies pour la ChAc est limitée par la rareté de cette maladie avec seulement quelques praticiens rencontrant plus d’un cas durant leur vie professionnelle [15]. La littérature est ainsi limitée à des cas cliniques ou à de petites séries de cas. Le traitement de la ChAc est uniquement symptomatique. Un traitement antiépileptique doit être démarré aussitôt après la première crise. Ceci s’explique par le risque accru de développer de nouvelles crises et par les complications associées aux crises tonico-cloniques (morsures de langue, accidents de trafic…). Les crises convulsives répondent généralement aux antiépileptiques standards comme la phenytoine et le valproate de sodium. La lamotrigine et le carbamazepine peuvent aggraver quant à eux les mouvements involontaires [9]. L´halopéridol est le médicament de choix pour s’attaquer aux mouvements choréiques. Enfin en règle générale les patients avec ChAc sont de mauvais candidat à la chirurgie de l’épilepsie [15].

## Conclusion

L’épilepsie peut précéder par des années le diagnostic d’une ChAc. Le praticien doit garder en mémoire l'éventualité de cette affection dans sa démarche diagnostique devant l’association de crises épileptiques et de mouvements anormaux.
